# Efficacy of an adenovirus-vectored foot-and-mouth disease virus serotype A subunit vaccine in cattle using a direct contact transmission model

**DOI:** 10.1186/s12917-018-1582-1

**Published:** 2018-08-29

**Authors:** John G. Neilan, Christopher Schutta, José Barrera, Melia Pisano, Laszlo Zsak, Ethan Hartwig, Max V. Rasmussen, Barbara J. Kamicker, Damodar Ettyreddy, Douglas E. Brough, Bryan T. Butman, David A. Brake

**Affiliations:** 1 U.S. Department of Homeland Security Science and Technology Directorate, Plum Island Animal Disease Center, P.O. Box 848, Greenport, NY 11944 USA; 2 The McConnell Group, Inc., Plum Island Animal Disease Center, P.O. Box 848, Greenport, NY 11944 USA; 3 Leidos, Plum Island Animal Disease Center, P.O. Box 848, Greenport, NY 11944 USA; 4 Plum Island Animal Disease Center Research Participation Program, Oak Ridge Institute for Science and Education, Oak Ridge, TN USA; 5 U.S. Department of Agriculture, Agricultural Research Service, Foreign Animal Disease Research Unit, Plum Island Animal Disease Center, P.O. Box 848, Greenport, NY 11944 USA; 6 GenVec, Inc., 910 Clopper Road, Suite 220N, Gaithersburg, MD 20878 USA; 7 BioQuest Associates, LLC, Plum Island Animal Disease Center, P.O. Box 848, Greenport, NY 11944 USA

**Keywords:** Foot-and-mouth disease virus, FMDV A24/Cruzeiro/BRA/55, Replication-deficient human adenovirus vectored vaccine, DIVA, Vaccine efficacy

## Abstract

**Background:**

A direct contact transmission challenge model was used to simulate natural foot-and-mouth disease virus (FMDV) spread from FMDV A24/Cruzeiro/BRA/55 infected ‘seeder’ steers to naïve or vaccinated steers previously immunized with a replication-deficient human adenovirus-vectored FMDV A24/Cruzeiro/BRA/55 capsid-based subunit vaccine (AdtA24). In two independent vaccine efficacy trials, AdtA24 was administered once intramuscularly in the neck 7 days prior to contact with FMDV A24/Cruzeiro/BRA/55-infected seeder steers.

**Results:**

In Efficacy Study 1, we evaluated three doses of AdtA24 to estimate the 50%/90% bovine protective dose (BPD_50/90_) for prevention of clinical FMD. In vaccinated, contact-challenged steers, the BPD_50/90_ was 3.1 × 10^10^ / 5.5 × 10^10^ AdtA24 particles formulated without adjuvant. In Efficacy Study 2, steers vaccinated with 5 × 10^10^ AdtA24 particles, exposed to FMDV A24/Cruzeiro/BRA/55-infected seeder steers, did not develop clinical FMD or transmit FMDV to other vaccinated or naïve, non-vaccinated steers. In contrast, naïve, non-vaccinated steers that were subsequently exposed to FMDV A24/Cruzeiro/BRA/55-infected seeder steers developed clinical FMD and transmitted FMDV by contact to additional naïve, non-vaccinated steers. The AdtA24 vaccine differentiated infected from vaccinated animals (DIVA) because no antibodies to FMDV nonstructural proteins were detected prior to FMDV exposure.

**Conclusions:**

A single dose of the AdtA24 non-adjuvanted vaccine conferred protection against clinical FMD at 7 days post-vaccination following direct contact transmission from FMDV-infected, naïve, non-vaccinated steers. The AdtA24 vaccine was effective in preventing FMDV transmission from homologous challenged, contact-exposed, AdtA24-vaccinated, protected steers to co-mingled, susceptible steers, suggesting that the vaccine may be beneficial in reducing both the magnitude and duration of a FMDV outbreak in a commercial cattle production setting.

## Background

Foot-and-mouth disease (FMD) afflicts cloven-hooved animals, including cattle, pigs, sheep, goats, and buffalo and is enzootic throughout most of Africa and Asia. In most susceptible animals, FMD is characterized by pedal and oronasal vesicular lesions. The causative agent, foot-and-mouth disease virus (FMDV), a *Picornaviridae* RNA virus*,* encodes capsid proteins, nonstructural proteins and proteases (reviewed [1]). There are seven FMDV serotypes, and numerous strains within each serotype that often fail to confer intra-serotype immunity following immunization. Many FMD endemic and epizootic countries currently control outbreaks through annual or semi-annual vaccination with conventional, inactivated vaccines. However, in FMD-free countries, next generation recombinant FMD vaccines produced without the use of virulent FMDV strains are more advantageous than inactivated vaccines, especially for a rapid response against newly emerging FMDV topotypes/viral lineages that are a poor antigenic match against current vaccines. For example, the FMDV capsid gene sequence from an outbreak strain can be obtained following virus isolation, rapidly synthesized, and inserted into a standardized viral-vector vaccine production platform. In the event of an outbreak in a FMD-free country, a ‘vaccinate to retain’ versus a ‘vaccinate to remove’ policy would benefit from a recombinant FMD subunit vaccine. The AdtA24 described below, based on the AdtFMD vaccine platform, is genetically deleted in antibody epitopes used in current FMD serological diagnostic tests and thus can differentiate infected from vaccinated animals (DIVA).

The FMDV main transmission route in nature is by aerosol or direct contact (reviewed [2, 3]). Numerous FMD inactivated vaccine studies demonstrating clinical FMD protection using indirect or direct challenge models in livestock have been reported (reviewed [4]). Cattle vaccinated with a conventional FMDV serotype O vaccine were assessed for clinical FMD and the ability to transmit FMDV following indirect co-housing with previously infected pigs for various times post-vaccination [5]. Results demonstrated that cattle vaccinated 21 days prior to challenge were protected against clinical FMD and failed to transmit FMDV to susceptible cattle. When the interval between vaccination and infected pig contact exposure was shortened, mixed results were observed, leading to the recommendation that in the event of an outbreak, FMD-vaccinated cattle should be sequestered from non-vaccinated cattle for a minimum of three weeks. Additional studies using FMD high potency vaccines and indirect aerosol challenge from infected pigs at 2–4 days post-vaccination (dpv) confirmed that vaccinated cattle and pigs were protected against clinical FMD [6, 7]. In a swine direct contact challenge model, when FMDV serotype O vaccinated pigs were directly exposed to infected pigs for only 2 h, the majority of vaccinated pigs developed clinical FMD [8]. Another study using vaccinated pigs followed by a 9 h direct challenge period reported similar findings [9]. Additional studies using vaccinated cattle directly exposed to FMDV-infected naïve cattle indicated that normal dose or high potency vaccinated cattle were fully protected 3 weeks post-vaccination following subsequent exposure to infected cattle for 5 days [10, 11]. However, protection was reduced to 70–75% when a shorter, 10 day vaccine-to-challenge interval was used [12].

A few studies have evaluated the effectiveness of next generation FMD vaccines in contact challenge models. Protection using adenovectored FMD-vaccinated pigs subsequently challenged by direct contact with infected pigs has been reported [13, 14]. We recently reported that a replication deficient, recombinant human adenovirus serotype 5 vectored (Ad5) adjuvant-free vaccine co-expressing the P1 capsid from FMDV A24/Cruzeiro/BRA/55 and 3C protease genes of FMDV A12/119/Kent/UK/32 (AdtA24) was efficacious at 7 dpv in cattle using a intradermolingual (IDL) direct challenge model [15] and enabled DIVA prior to challenge. This AdtA24 vaccine also passed five safety evaluations: no adverse effects on calves, no reversion to virulence, no shedding from vaccinees to naïve animals, no excretion in milk from lactating dairy cows, and < 4% transient injection site reactions in 500 beef and dairy cattle evaluated under field conditions [16]. In order to expand these initial studies, we assessed AdtA24 vaccine efficacy in cattle using two different direct contact FMDV challenge experimental designs.

## Methods

### Animals

Healthy Holstein cross-bred steers, three to six months of age and 130–230 kg were purchased from an Association for the Assessment and Accreditation of Laboratory Animal Care accredited livestock facility. Animal care and study conduct were in compliance with the guidelines of and approved by the Plum Island Animal Disease Center (PIADC) Institutional Biosafety Committee and the Institutional Animal Care and Use Committee. Steers were acclimated and housed in the PIADC BSL-3 Ag animal facility. Prior to vaccination, steers were randomly allocated to treatment groups.

### Experimental FMD vaccine

The AdtA24 vaccine vector was produced by GenVec, Inc. (Gaithersburg, MD) as previously summarized [15, 17, 18]. AdtA24 contains the P1-2A coding regions from FMDV A24/Cruzeiro/BRA/55, and the partially deleted (missing the amino terminus six amino acids) 3B1, complete 3B2, 3B3, and 3C nonstructural protein coding regions from FMDV A12/119/Kent/UK/32. For each vaccine lot, total particle units (PU) were quantified by HPLC [17], and all vaccines were stored at -80 °C. On the day of vaccination, thawed vaccines were diluted in final formulation buffer (FFB; Lonza). No adjuvants were used in these studies and for each study, baseline serum samples were collected immediately prior to vaccination (Day 0). Steers were inoculated intramuscularly (IM) in the cleidooccipitalis muscle with a single 2 ml injection with placebo (FFB) for control animals or one of the AdtA24 vaccine doses listed below for each study.

### Challenge virus preparation and administration

FMDV A24/Cruzeiro/BRA/55 challenge virus (1 × 10^4^ bovine infectious dose 50% /0.4 mL) was prepared and administered by the intradermal lingual (IDL) route to naïve control or naïve ‘seeder’ steers. Each steer received four, 0.1 ml injections in the tongue, as described previously [15, 18].

### Experimental study designs

#### Efficacy study 1

**Fig. 1:**
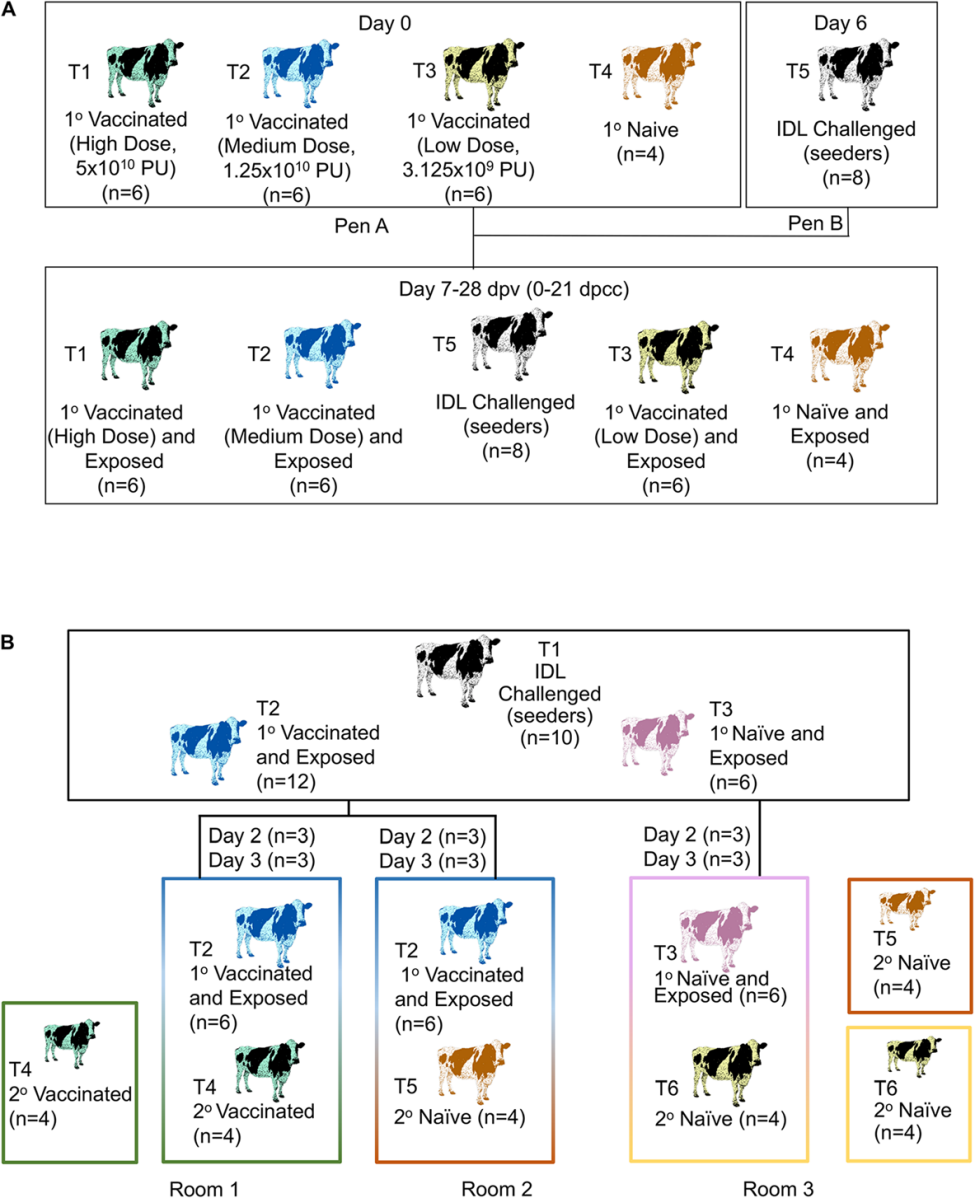
**a**. Efficacy Study 1 Experimental Design: Evaluation of vaccinated and non-vaccinated steers following contact with FMDV-infected steers. Top row: A total of 18 steers vaccinated with either a high, medium or low dose of AdtA24 vaccine and 4 naïve steers were co-mingled beginning 7 dpv with 8 steers that were directly infected with FMDV 24 h earlier (6 dpv). **b**. Efficacy Study 2: Experimental Design: Evaluation of vaccinated and non-vaccinated steers in contact with FMDV-exposed steers. Top row: 12 “primary (1^o^) vaccinated and exposed” T2 steers were vaccinated with 5 × 10^10^ particle units of AdtA24 7 days prior to co-mingling with 10 T1 steers that were infected with FMDV via the intradermolingual (IDL) route on Day 0. The 10 T1 IDL-challenged ‘seeder’ steers were mingled with 12 T2 AdtA24 “1^o^ vaccinated and exposed” steers for 2 (*n* = 6 steers) and 3 days (*n* = 6 steers) and with 6 T3 “1^o^ naïve and exposed” steers for 2–3 days (3 steers for each day of duration). Bottom rows: for the next 41 days, 4 T4 2^o^ vaccinated + 6 T2 1^o^ vaccinated and exposed steers were in room 1; 4 T5 2^o^ naïve + 6 T2 1^o^ vaccinated and exposed steers were in room 2; 4 T6 2^o^ naïve + 6 T3 1^o^ naïve steers were in room 3

The objective was to determine the AdtA24 immunogenic properties of three different vaccine doses and to evaluate their efficacy to protect cattle against experimental FMDV contact challenge at 7-days post-vaccination. Six steers per group were allocated for T1, T2 and T3, four steers to T4, and eight ‘seeder’ steers to T5 (Fig. 1a**)**. AdtA24 treatments groups were: T1, 5 × 10^10^ PU (High dose); T2, 1.25 × 10^10^ PU (Medium dose); T3, 3.1 × 10^9^ PU (Low dose). Primary (1^o^) Naïve, contact-challenged steers (T4) received FFB. Following injections, T1-T4 steers were co-mingled in the same pen and room. On Day 6, T5 ‘seeder’ steers were infected via the IDL route, housed for 24 h in a separate pen in the same room housing T1-T4 steers, and then all 30 steers were allowed to intermingle starting on Day 7 (0 dpcc). The ratio of vaccinated (T1-T3) to infected steers (T5) was 18:8 (2.25:1) during 0–2 dpcc, Once the naïve contact (T4) and infected steers (T5) developed full clinical disease (pedal lesions on all 4 hooves)(3–6 dpcc) T4-T5 steers were from the co-mingling pen to another pen located in the same room through study durations (21 dpcc). Serum samples were collected weekly for the virus neutralization test starting on the day of vaccination (prior to AdtA24 administration). Oral and nasal swabs (Dacron polyester) were collected on 0–5 dpcc to detect the presence of FMDV by rRT-PCR.

#### Efficacy study 2

The main objective was to determine if a single high (emergency) dose of AdtA24 could prevent primary vaccinated ‘donor’ cattle contact exposed to FMDV infected cattle, from subsequent FMDV transmission to non-vaccinated, naïve cattle. On Day − 7, twelve T2 1^o^ steers were vaccinated with 5 × 10^10^ PU AdtA24 and six T3 1^o^ naïve steers were injected with a placebo (FFB). Between Day − 7 and Day − 1, the T2 1^o^ and T3 1^o^ naïve steers were co-mingled with ten T1 ‘seeder’ steers which were IDL challenged with A24/Cruzeiro/BRA/55 on Day 0. (Fig. 1b). After 2 days post contact challenge (2 dpcc) by the 10 T1 IDL-challenged seeder steers: (i) three T2 1^o^ vaccinated and exposed steers were moved into Room 1 that housed T4 AdtA24 secondary (2^o^) vaccinated steers (*n* = 4) previously immunized on Day − 5, (ii) three T2 1^o^ vaccinated and exposed steers were moved into Room 2 that housed four T5 non-vaccinated (2^o^ naïve) steers, and (iii) three T3 placebo-injected (1^o^ naïve and exposed) steers were moved into Room 3 that housed four T6 recipient non-vaccinated (2^o^ naïve) steers. This same allocation method was repeated 24 h later (after 3 dpcc) with the remaining six T2 1^o^ vaccinated and exposed steers and three T3 placebo-injected (1^o^ naïve and exposed) steers. Steers were then co-mingled within each room (10 steers/room; Rooms 1–3) for an additional 36 days (study termination). For the initial study phase during 0–2 dpcc, the ratio of donor vaccinated to naïve steers was 0.75:1, and decreased to 0.46:1 on 2–3 dpcc. For the subsequent 4–43 dpcc study phase, the ratio of T2 donor vaccinates to T4 recipient vaccinates or T5 recipient naïve steers was 1:1. Serum samples were collected weekly for the virus neutralization test and to detect antibodies to the FMDV nonstructural proteins starting on the day of vaccination (prior to AdtA24 administration). Plasma samples were collected daily on 0–7 and 9 dpcc. Oral (mouth) and nasal samples were separately collected on 0, 2, and 4 dpcc, and probang samples on 29–30, 36–37, and 42–43 dpcc to detect the presence of FMDV by rRT-PCR.

### Clinical observations and analytical assays

Trained scientists, through masked treatment allocation, performed clinical observations (lesions). Following IDL challenges, the presence or absence of clinical FMD in sedated steers was assessed twice per week through the study termination date. FMD clinical signs and lesions used the following criteria: negative, no pedal or oronasal (lip, mouth or nose) vesicular lesions; positive, one or more pedal or oronasal vesicular lesions.

### Virus neutralization test (VNT)

Beginning on the day of vaccination, serum samples were collected weekly, prior to administration of any treatments, heat inactivated (56 °C, 30 min), and stored at -20 °C. FMDV A24/Cruzeiro/BRA/55 VNT titers were determined on BHK21 [C13] (ATCC® CCL10™) cells, measuring cytopathic effect, as previously described [15]. The VNT geometric mean titers (GMT) were calculated using a value of 0.6 log_10_ for samples that were below the limit of detection. A test sample was scored positive if the VNT titer was ≥0.9 log_10_.

### Detection of FMDV or FMDV nucleic acid

#### Clinical samples

LFBKα_v_β_6_ cells (kindly provided by M. LaRocco, USDA Agricultural Research Service, PIADC [19, 20]) were used to detect FMDV from plasma, oral, nasal, and probang samples based on cytopathic effect [18]. Oral and nasal swabs were placed separately into chilled transport medium, mixed, removed, and samples frozen at -70 °C. Thawed, centrifuged, and clarified samples (Spin-X centrifuge tube filters) were tested. FMDV nucleic acid in plasma, oral, and nasal samples was detected by Real-Time Reverse Transcriptase-Polymerase Chain Reaction (rRT-PCR) [18, 21]. A sample C_t_ value < 40 was scored as positive. Oral and nasal results are reported together (i.e., oronasal fluids).

#### Air samples

In Efficacy Study 2, to detect circulating FMDV nucleic acid by rRT-PCR, two air filter samples were collected on opposite sides of each room for all three rooms daily for 18 days (2–20 dpcc) and then on alternate days until 43 dpcc. Air sampling was performed as previously described [22] and filters were replaced every 24 h. A sample was considered positive if the Ct value was < 40 in at least one air filter/room. Samples for each room were tested separately and results are shown for each room.

### Detection of antibodies to FMDV non-structural proteins (NSP)

During both studies, weekly serum samples were collected to detect antibodies to FMDV 3ABC NSPs using the PrioCHECK® FMDV NS ELISA (ThermoFisher Scientific), according to manufacturer’s instructions. A serum sample was considered positive if the percent inhibition was ≥50%.

### Data analysis

Serum VNT geometric mean titers and standard deviations were calculated (Microsoft Excel). Comparisons between treatments within an experiment were done using unpaired one- or two-tailed T-tests (Excel). *p-*values ≤0.05 were considered significant. BPD_50_ and BPD_90_ values were calculated by the Spearman-Kärber method using lesion data obtained on 14 dpcc [23].

## Results

### Efficacy – Study 1

On the day of contact challenge (7 dpv/0 dpcc), 100%, 100%, and 67% of T1-T3 vaccinates, respectively had FMDV A24/Cruzeiro/BRA/55 VNT titers (Table 1**)**. VNT responses were vaccine dose-dependent, and the T1 GMT (2.2 ± 0.2) was statistically higher compared to T2 (1.5 ± 0.5) (*p* = 0.01) and T3 (1.1 ± 0.5) (*p* < 0.001). On 3 days post-challenge, all eight IDL-infected seeder steers (T5) had pedal lesions on all four hooves except for one with three hooves with lesions, and all had oronasal lesions. Two of four 1^o^ naïve contact controls (T4) developed pedal lesions by 3dpcc: one had two and the other had four hooves affected. On 7 dpcc, all four T4 steers were positive on all hooves. In T1-T3 vaccinates, protection against clinical FMD (e.g., any vesicular lesion) was vaccine dose-dependent, since 50% of T1 high dose vaccinates were completely protected compared to 33% and 0% of T2, medium dose, and T3, low dose, vaccinates. The BPD_50/90_ for prevention of clinical FMD (any lesions) was 3.1 × 10^10^ / 5.5 × 10^10^ PU. All vaccinates were rRT-PCR positive for FMDV RNA in the oronasal cavity. The majority (16/18; 89%) of AdtA24 vaccinated steers developed NSP antibodies at 14 dpcc (Table 1). The two NSP negative, vaccinated steers in the T1 high dose group were also completely protected against pedal and oronasal lesions (data not shown).

**Table 1:** Efficacy Study 1. Summary of outcomes based on clinical and laboratory results

Treatment Group	N	Percent Protected from Lesions (21 dpcc)		Geometric mean FMDV A24/Cruzeiro/BRA/55 VNT titer (log_10_) ± SD on 7 dpv/0 dpcc (range)	Percent Positive for FMDV A24/Cruzeiro/BRA/55 VNT on 7 dpv/0 dpcc	Percent Protected from FMDV RNA in oral and nasal cavities (0–5 dpcc)	Percent Positive for FMDV NSP Antibodies
		Pedal only	Pedal and Oronasal				
T1: 1^o^ vaccinated AdtA24 5 × 10^10^ PU (high dose) Contact Challenge	6	83%	50%	2.2 ± 0.2^a,b^ (1.8–2.4)	100%	0%	0% - 0 dpcc 67% - 14 dpcc
T2: 1^o^ vaccinated AdtA24 1.25 × 10^10^ PU (medium dose) Contact Challenge	6	100%	33%	1.5 ± 0.5^a^ (0.9–2.1)	100%	0%	0% - 0 dpcc 100% - 14 dpcc
T3: 1^o^ vaccinated AdtA24 3.125 × 10^9^ PU (low dose) Contact Challenge	6	67%	0%	1.1 ± 0.5^b^ (0.6–1.8)	67%	0%	0% - 0 dpcc 100% - 14 dpcc
T4: 1^o^ naïve Contact Challenge (control)	4	0%	0%	0.6 ± 0.0*	0%	0%	ND
T5 (IDL challenged seeder steers) 1 day prior to contact with T1-T4	8	0%	0%	0.6 ± 0.0*	0%	0%	ND

*Dpcc* days post contact challenge, *DPV* days post vaccination, *VNT* virus neutralization test, *1*^*o*^ primary, *PU* particle units, *IDL* intradermolingual challenge, *ND* Not Determined

^*^A positive VNT is ≥0.9 log_10_. ^a,^ *p* = 0.01 for T1 > T2. ^b,^ *p* = < 0.001 for T1 > T3. *p* = 0.1 for T2 and T3

### Efficacy – Study 2

On 7 dpv (day of contact challenge with 10 T1 IDL challenged ‘seeder’ steers with lesions on all hooves), 100% of T2 1^o^ vaccinated and exposed steers had FMDV VNT titers (GMT = 1.5 ± 0.2 log_10_). Additionally, 75% (3/4) of T4 AdtA24 2^o^ vaccinates (Room 1) had FMDV VNT titers (GMT = 0.9 ± 0.2 log_10_) at 5 dpv, the first day of contact challenge with T2 1^o^ vaccinated and exposed steers (Table 2).

**Table 2:** Efficacy Study 2: Summary of FMDV Geometric mean virus neutralization titers (GMT) in cattle

Treatment Group	FMDV A24/Cruzeiro/BRA/55 GMT (± std. dev.; log_10_)			
	Days Post-Vaccination/Post Contact Challenge (% seropositive)			
	7/0	14/7	21/14	24/17
12 T2: 1^o^ Vaccinated and Exposed to 10 IDL challenged seeder cattle for 2–3 days	1.5 ± 0.2** (100%)	1.7 ± 0.2 (100%)	2.0 ± 0.4** (100%)	1.9 ± 0.2** (100%)
6 T3: 1^o^ naïve and Exposed to 10 IDL challenged seeder cattle for 2–3 days	0.6 ± 0.0 (0%)	1.4 ± 0.5 (83%)	2.6 ± 0.2 (100%)	2.8 ± 0.4 (100%)
	FMDV A24/Cruzeiro/BRA/55 GMT (± std. dev.; log_10_)			
	Days Post-Vaccination/Post Contact Challenge (% seropositive)			
	5/−2	12/5	19/12	22/15
4 T4: 2^o^ Vaccinated; Intermingled with 6 T2: 1^o^ Vaccinated and Exposed cattle in Room 1 for 35 days	0.9 ± 0.2* (75%)	1.5 ± 0.4** (100%)	1.3 ± 0.2** (100%)	1.7 ± 0.4** (100%)
4 T5: 2^o^ naïve; Intermingled with 6 T2: 1^o^ Vaccinated and Exposed cattle in Room 2 for 35 days	0.6 ± 0.0 (0%)	0.6 ± 0.0 (0%)	0.6 ± 0.0 (0%)	0.6 ± 0.0 (0%)
4 T6: 2^o^ naïve; Intermingled with 6 T3: 1^o^ naïve and Exposed cattle in Room 3 for 35 days	0.6 ± 0.0 (0%)	0.6 ± 0.0 (0%)	2.0 ± 0.2** (100%)	2.9 ± 0.3** (100%)

All are two-tailed T-test comparisons within the specified time period

Vaccinated cattle received 5 × 10^10^ particle units of AdtA24

*1*^*o*^ primary, *2*^*o*^ secondary, *IDL* intradermolingual challenge

^*^different at *p* = 0.05; ^**^different at *p* ≤ 0.01

All ten T1 IDL challenged seeder steers developed clinical disease by four days post challenge (Table 3). All six T3 1^o^ naïve contact-exposed steers developed clinical FMD by 7 dpcc with lesions on all hooves, and all had oronasal lesions. In addition, each T3 1^o^ naïve contact-exposed steer had at least one FMDV-positive plasma and oronasal fluid sample during the first five dpcc. Similarly, all four T6 2^o^ naïve steers co-mingled with T3 1^o^ naïve and contact-exposed steers developed clinical FMD by 7 dpcc and had at least one FMDV-positive plasma and oronasal fluid sample by 9 dpcc.

**Table 3:** Efficacy Study 2. Summary of protection based on clinical and laboratory results

Treatment Group		Percent Protection from Outcomes					Percent Positive for Antibodies to FMDV NSPs
	N	Clinical FMD (1–15 dpcc)	FMDV or FMDV RNA in plasma (0–7, 9 dpcc)	FMDV RNA in oronasal fluid (0, 2, 4 dpcc)	FMDV RNA in probang (27, 34, 41 dpcc)	FMDV in probang (27, 34, 41 dpcc)	
T1: IDL challenged seeder steers	10	0%	ND	ND	ND	ND	ND
T2: 1^o^ AdtA24 5 × 10^10^ PU vaccinated; Intermingled with 10 IDL challenged ‘seeder steers’ for 2–3 days	12	100% No oronasal lesions	100%	0%	18%	27%	9%^a^ - 0 dpcc 91% -30 dpcc
T3: 1^o^ naïve; Intermingled with 10 IDL challenged ‘seeder steers’ for 2–3 days	6	0%	0%	0%	17%	17%	0% - 0 dpcc 100%-30 dpcc
T4: 2^o^ AdtA24 5 × 10^10^ PU vaccinated; Intermingled with 6 T2: 1^o^ vaccinated and exposed steers (Room 1)	4	100% No oronasal lesions	100%	100%	100%	100%	0% - 0 and 30 dpcc
T5: 2^o^ naïve; Intermingled with 6 T2: 1^o^ vaccinated and exposed steers (Room 2)	4	100% No oronasal lesions	100%	75%	50%	100%	0% - 0 and 30 dpcc
T6: 2^o^ naïve; Intermingled with 6 T3: 1^o^ naïve and exposed steers (Room 3)	4	0%	0%	0%	25%	25%	0% - 0 dpcc 100%-30 dpcc

*Dpcc* days post contact challenge, *NSP* nonstructural protein, *1*^*o*^ primary, *2*^*o*^ secondary, *IDL* intradermolingual challenge, *PU* particle units

^a^one false positive (consistent with reported diagnostic specificity rates for this assay [36, 37])

In contrast, all 12 of the AdtA24 T2 1^o^ vaccinated and contact-exposed steers were completely protected against clinical FMD and viremia (Table 3). However, each T2 1^o^ vaccinated and exposed steer had at least one PCR-positive FMDV oronasal fluid sample at either 2 or 4 days post-contact exposure to the 10 T1 IDL challenged ‘seeder’ steers. In addition, 73% of T2 1^o^ vaccinated and exposed steers had at least one FMDV-positive probang sample following contact exposure to the 10 T1 IDL challenged ‘seeder’ steers.

All T4 2^o^ vaccinated and exposed steers were completely protected against clinical FMD, viremia, oronasal infection and remained probang-negative following contact exposure to T2 1^o^ vaccinated and contact-exposed steers (Table 3). Notably, following co-mingling with T2 1^o^ vaccinated and contact-exposed steers, none of the four T5 2^o^ naïve steers became infected, as evidenced by the absence of clinical FMD, viremia, and FMDV positive probang samples.

**Fig. 2:**
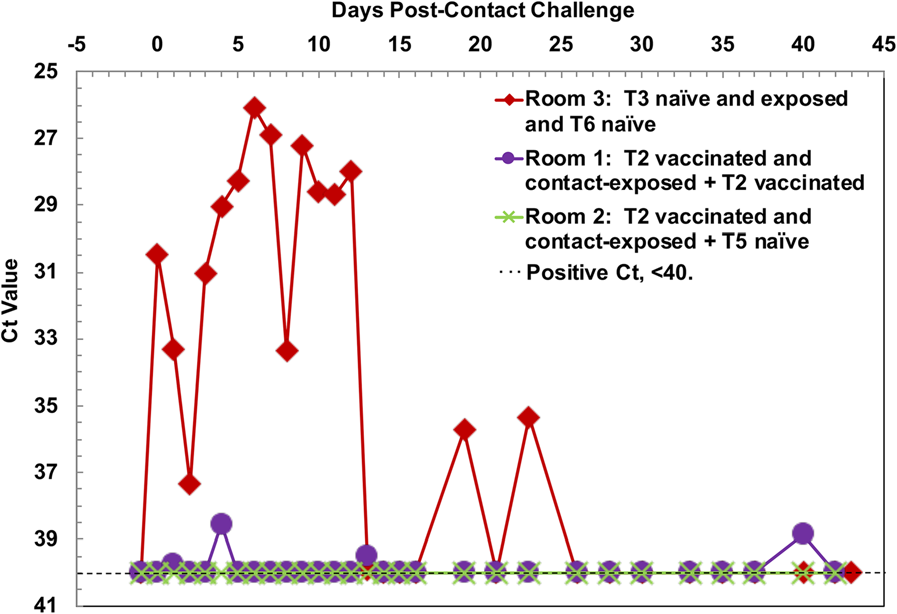
Efficacy Study 2: Detection of FMDV nucleic acid in air filters collected from animal rooms. FMDV nucleic acid in air samples that were collected on air filters from each of the three animal rooms, were detected by rRT-PCR, and a C_t_ value < 40 was scored positive. Air filters were collected daily for the first 18 days and then on alternate days to 43 days. The experimental design is diagramed in Fig. 1b

All 27 Room 2 air samples collected during the secondary contact exposure study phase with all four T5 2^o^ naïve and six T2 1^o^ vaccinated and contact-exposed steers were FMDV negative by PCR (Fig. 2). In Room 1 containing all four T4 2^o^ vaccinated and exposed steers and six T2 1^o^ vaccinated and contact-exposed steers, 86% of 28 air samples collected during this same time period were PCR negative, and the remaining 14% were borderline positive (Ct = 38.5- < 40). In contrast, 48% of 28 Room 3 air samples were PCR positive; none of the steers were vaccinated: four T6 2^o^ naïve steers and six T3 1^o^ naïve contact-exposed steers.

Eleven of twelve (91%) of T2 1^o^ vaccinated and contact-exposed steers, although fully protected against clinical FMD, developed FMDV NSP antibodies at 30 dpcc (Table 3). In contrast, during this same period, no T4 2^o^ vaccinated and contact-exposed, protected steers or T5 2^o^ naïve, protected steers were NSP antibody positive following co-mingling with T2 1^o^ vaccinated and contact-exposed steers. All four T6 2^o^ naïve, non-protected steers in Room 3 developed FMDV NSP antibodies following 43 days co-mingling with T3 1^o^ naïve contact-exposed steers.

## Discussion

The ability of a non-adjuvanted AdtA24 recombinant subunit vaccine to confer protection against clinical FMD in immunized cattle at 7 dpv following subsequent direct contact exposure to previously FMDV-infected, naïve ‘seeder’ steers was assessed in two independent studies. Collectively across all vaccine doses tested, the AdtA24 vaccine conferred 62% protection (21 fully protected/34 vaccinated, FMDV exposed) against clinical FMD. The higher protection against clinical FMD observed in the second efficacy study (100%) compared to the first efficacy study (50%) with the highest vaccine dose (5 × 10^10^ PU) may be due to AdtA24 vaccine lot differences and the presence of non-neutralizing antibody antigenic vaccine components (i.e., pentamers) produced in AdtA24 virus vaccine production and purification in the second lot. These two studies also show AdtA24 vaccine DIVA capability prior to contact exposure. However, additional studies designed to validate the AdtA24 DIVA attribute under repeated vaccination and sampling at longer time points post-vaccination are necessary.

In Efficacy Study 1, on 7dpv/0 dpcc, 89% (16/18) of T1-T3 immunized steers had vaccine dose-dependent FMDV VNT titers, with the statistically highest GMT in the highest dose group (T1) (Table 1**)**. The estimated BPD_50/90_ of 3.1 × 10^10^/5.5 × 10^10^ PU against any vesicular lesions in this study was comparable to results obtained in steers vaccinated with AdtA24 and directly infected intradermolingually 7 days later with FMDV A24 (BPD_50/90_ to prevent clinical FMD was 1.0 × 10^10^/5.6 × 10^10^ PU) [15]. Since an AdtA24 vaccine formulated in ENABL® adjuvant significantly lowered the vaccine protective dose in the IDL direct challenge model [18], future studies should be conducted to determine if an AdtA24/ ENABL® formulation can also lower the BPD_50/90_ values reported in this study.

In Efficacy Study 2 (Fig. 1b), a more complex study design was used to evaluate the ability of direct contact challenge exposed AdtA24 primary vaccinates to transmit FMDV to produce clinical or subclinical FMD to other AdtA24 secondary vaccinates as well as to naïve, non-vaccinated cohorts. We acknowledge the limitations in this laboratory research experimental design for Efficacy Study 2, specifically the tightly controlled, very high air exchange rate, and intentional animal movement/room re-distribution compared to the natural feedlot, pasture or dairy parlor setting. The onset of clinical FMD observed in the non-vaccinated steers in contact with FMDV-infected steers was consistent with the 1–6 day onset reported in a previous study [24]. Under the research conditions and experimental design used herein, the contact transmission model clearly demonstrated that naïve, non-vaccinated steers with active clinical FMD readily transmitted FMDV to other naïve, non-vaccinated steers, resulting in clinical FMD. AdtA24 vaccinated steers failed to develop clinical FMD. Notably, despite evidence of FMDV in AdtA24 1^o^ vaccinated steers’ oronasal fluid and probang samples, these steers did not transmit clinical or subclinical FMD to AdtA24 2^o^ vaccinated steers or to 2^o^ naïve, non-vaccinated steers, all of which remained FMDV seronegative by VNT and NSP antibody assays. Air samples collected for 43 days following co-mingling of AdtA24 1^o^ vaccinated, contact-exposed steers with 2^o^ naïve non-vaccinated steers were consistently negative, or in the case of the room with AdtA24 2^o^ vaccinated steers, were sporadically borderline positive. The rRT-PCR values obtained from air samples in rooms with infected cattle were consistent with the values reported in another cattle study during 0–12 dpcc [25]. The consistently lower Ct values in room 3 compared to room 1 suggest that the AdtA24 vaccine may help reduce the FMD virus environment load during a natural outbreak. Results also support the conclusion that detection of rRT-PCR-positive oronasal or probang samples at approximately 4–6 weeks post-contact exposure in these disease-free ‘carrier’ AdtA24-vaccinated, donor steers is unlikely to play a role in FMDV transmission or FMD outbreak control efforts. The epidemiological importance of persistently infected cattle (“carrier state”) remains a controversial topic for discussion and further research [26, 27]. However, our results are consistent with conclusions drawn by other FMD researchers [28, 29].

Based on our results, we expect that AdtA24-vaccinated cattle would be protected from contact transmission of FMDV from other FMDV-infected hosts, such as sheep, pigs, goats, and buffalo. A variety of studies have demonstrated the effectiveness of other types of FMDV vaccines in preventing transmission of FMDV among animals [5, 10–12, 14, 30–35].

## Conclusion

In summary, using a simulated natural FMDV infection route, we demonstrated that the replication-deficient AdtA24 FMD DIVA vaccine was effective at preventing clinical FMD and viremia following direct contact transmission exposure. Under field conditions following a FMD outbreak in a previous FMD-free country, we expect that AdtA24 and other AdtFMD serotype/subtype DIVA vaccines will be effective in reducing FMDV transmission from vaccinated to naïve steers, leading to a reduction in both disease outbreak magnitude and duration.

## Abbreviations

AdtA24: Replication-deficient human adenovirus vectored FMDV A24/Cruzeiro/BRA/55 vaccine
BPD_50/90_: Estimated 50%/90% bovine protective dose
Ct: Cycle threshold
DIVA: Differentiate infected from vaccinated animals
dpcc: Days post contact challenge
dpv: Days post-vaccination
FFB: Final formulation buffer
FMD: Foot-and-mouth disease
FMDV: Foot-and-mouth disease virus
GMT: Geometric mean virus neutralization test titer
IDL: Intradermolingual
NSP: Nonstructural protein
PIADC: Plum Island Animal Disease Center
PU: Particle units
rRT-PCR: Real-Time Reverse Transcriptase-Polymerase Chain Reaction
USDA: United States Department of Agriculture
VNT: Virus neutralization test
